# Airpart: interpretable statistical models for analyzing allelic imbalance in single-cell datasets

**DOI:** 10.1093/bioinformatics/btac212

**Published:** 2022-04-06

**Authors:** Wancen Mu, Hirak Sarkar, Avi Srivastava, Kwangbom Choi, Rob Patro, Michael I Love

**Affiliations:** Department of Biostatistics, University of North Carolina-Chapel Hill, Chapel Hill, NC 27514, USA; Department of Biomedical Informatics, Harvard Medical School, Boston, MA 02115, USA; New York Genome Center, New York, NY 10013, USA; The Jackson Laboratory, Bar Harbor, ME 04609, USA; Department of Computer Science, University of Maryland, College Park, MD 20742, USA; Department of Biostatistics, University of North Carolina-Chapel Hill, Chapel Hill, NC 27514, USA; Department of Genetics, University of North Carolina-Chapel Hill, Chapel Hill, NC 27514, USA

## Abstract

**Motivation:**

Allelic expression analysis aids in detection of *cis*-regulatory mechanisms of genetic variation, which produce allelic imbalance (AI) in heterozygotes. Measuring AI in bulk data lacking time or spatial resolution has the limitation that cell-type-specific (CTS), spatial- or time-dependent AI signals may be dampened or not detected.

**Results:**

We introduce a statistical method *airpart* for identifying differential CTS AI from single-cell RNA-sequencing data, or dynamics AI from other spatially or time-resolved datasets. *airpart* outputs discrete partitions of data, pointing to groups of genes and cells under common mechanisms of *cis*-genetic regulation. In order to account for low counts in single-cell data, our method uses a Generalized Fused Lasso with Binomial likelihood for partitioning groups of cells by AI signal, and a hierarchical Bayesian model for AI statistical inference. In simulation, *airpart* accurately detected partitions of cell types by their AI and had lower Root Mean Square Error (RMSE) of allelic ratio estimates than existing methods. In real data, *airpart* identified differential allelic imbalance patterns across cell states and could be used to define trends of AI signal over spatial or time axes.

**Availability and implementation:**

The *airpart* package is available as an R/Bioconductor package at https://bioconductor.org/packages/airpart.

**Supplementary information:**

Supplementary data are available at *Bioinformatics* online.

## 1 Introduction

Measurement of allelic expression (AE) through RNA-sequencing experiments can be used to detect genes for which genetic variation in local *cis*-regulatory elements (CRE) affects cell, tissue and organism development. Allelic imbalance (AI), in which one allele is expressed higher or lower than the other, may indicate local CRE where regulatory function, e.g. binding of a transcription factor to its motif, is impacted by genetic variation. AI could also reflect allelic differences in epigenetic state, as in the case of imprinting where maternal or paternal inheritance determines which allele is expressed higher, or genetic variation affecting splicing or nonsense mediated decay. When AE is quantified in bulk tissue or in a manner lacking the necessary time or spatial resolution, cell-type-specific (CTS) or contextual AI signals may be weakened. As the catalog of accessible CRE and active transcription factors differs across cell lineage, developmental time and spatial location (Heinz *et al.*, 2015), single-cell, temporal and spatial transcriptomic datasets can help to reveal the cell type, cell state or spatial dependencies of genetic effects (Andergassen *et al.*, 2017; Combs and Fraser, 2018; Wills *et al.*, 2013). For example, it has been observed that allele imbalance changes dynamically along embryo development stage (Deng *et al.*, 2014; Larsson *et al.*, 2019) and at human leukocyte antigen (HLA) genes and other autoimmune loci (Gutierrez-Arcelus *et al.*, 2020).

AE analysis cannot detect all variants detectable from expression quantitative trait loci (eQTL) analysis, which examines the association of total expression with genotype, as AE analysis is restricted to those genes and individuals that harbor heterozygous exonic variants (Khansefid *et al.*, 2018). However, as total expression level can be affected by technical artifacts (batch effects), environmental effects or distal-regulation, the within-individual comparisons in AE analysis offers an advantage in focusing on *cis*-regulatory effects, and may increase power (Vigorito *et al.*, 2021). Single-cell studies offer a unique opportunity to detect extra *cis*-eQTLs that would not have been identified in bulk, and hence a number of single-cell studies have been proposed to identify CTS *cis*-eQTL (Cuomo *et al.*, 2021a, b; Van Der Wijst *et al.*, 2018). Perhaps due to difficulties in obtaining single-cell AE with sufficient coverage, less attention has been paid to single-cell AE analysis, though single-cell AE analysis can be performed even within a single sample, while single-cell eQTL requires a population of cells of different genotype. Recent SMART-Seq2 and SMART-Seq3 experiments enable full-length transcript coverage from single cells at sufficient unique molecular depth to characterize AE for over 10 000 genes. When applied to cells of F1 offspring from crosses of different strains or species, AE data can be generated across hundreds or thousands of cells (Hagemann-Jensen *et al.*, 2020; Larsson *et al.*, 2019; Picelli *et al.*, 2014).

Prior studies in single-cell AE have categorized genes by allelic state (e.g. bi- or mono-allelic to one or the other allele), estimated allele-specific burst kinetics (Jiang *et al.*, 2017) and resolved multi-mapping reads to genes and alleles in order to reduce spurious mono-allelic signal (Choi *et al.*, 2019). In this work, we do not address the problem of accurate estimation of AE, assuming access to long reads uniquely assigned to alleles as obtained with SMART-Seq or similar technologies (Tian *et al.*, 2021). Previous methods have been proposed to detect imprinted genes from single-cell AE (Santoni *et al.*, 2017), whereas we focus on detection of CTS *cis*-genetic regulation resulting in a consistent imbalance within a group of cells toward a particular allele regardless of parent-of-origin. Furthermore, a recent regression-based method has been proposed to leverage datasets with bulk AE and single-cell total expression of the same tissue, to infer CTS AI (Fan *et al.*, 2021). Here, we examine single-cell AE datasets, as well as other contextually resolved AE data, including spatial or time course AE. When the AI only exists in one or more specific cell type(s) or the AI varies among cell types, we refer to this phenomenon as differential allelic imbalance (DAI). As single-cell studies providing sufficient coverage and read length for allelic quantification are only now emerging, we are aware of only one related statistical method for detecting DAI, *scDALI* (Heinen *et al.*, 2022), which models allele-specific chromatin accessibility using Gaussian Process regression.

Here, we introduce *airpart*, an AI R package for PARTitioning groups of cells, leveraging methods for the Generalized Fused Lasso (GFL) (Devriendt *et al.*, 2021) and hierarchical Bayesian modeling, to identify DAI across groups of cells or samples. Our AI models are flexible in terms of the experimental design, and can be applied to group cells or samples by cell type, spatial location or time points, as well as allowing adjustment for covariates. The gene clustering and partitioning of cell types by similar AI signal increases accuracy in the subsequent AE estimation step, alleviating issues from low counts and small numbers of cells for certain cell types or cell states.

Our method helps to find subsets of genes sharing similar DAI signals and helps to generate hypotheses of CTS *cis*-regulatory mechanisms, which can be further validated through experimentation assaying CRE activity or accessibility in particular cell types. The method is available as an R/Bioconductor (Huber *et al.*, 2015) package with an accompanying software vignette at https://bioconductor.org/packages/airpart.

## 2 Materials and methods

**Fig. 1.**
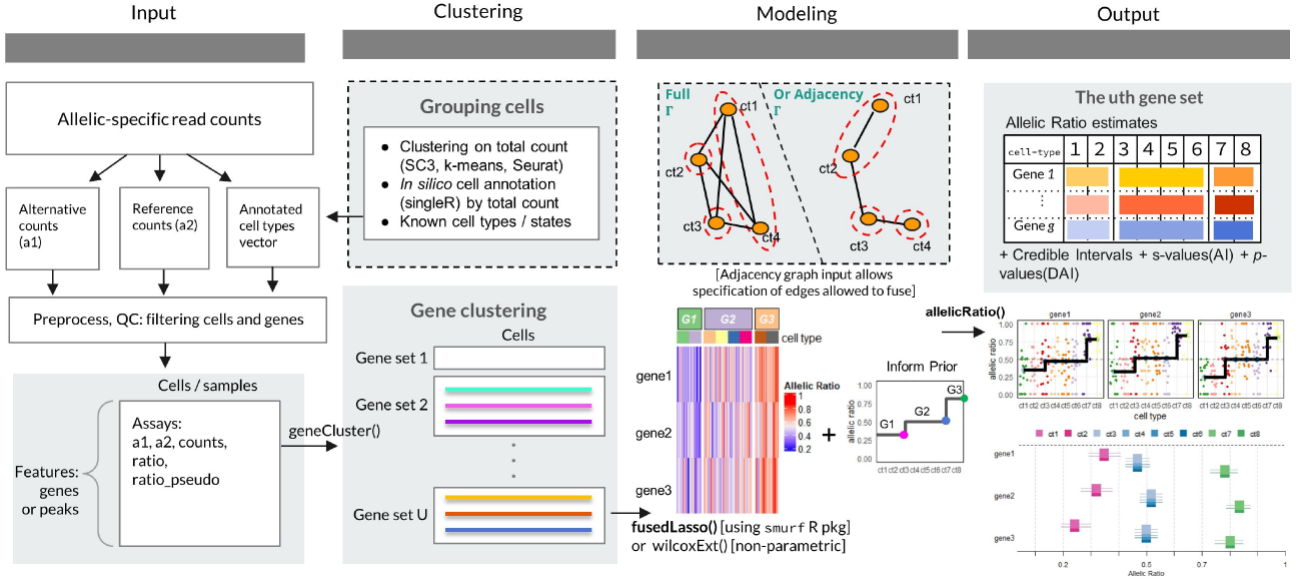
Overview of *airpart* framework. *airpart* takes as input allele-specific read counts, quantified upstream of our method. Known cell annotation or cell clusters derived from total counts are also part of the input to *airpart*. Following QC steps, clustering is performed on genes based on their allelic signal over cells. Then during the modeling step, a partition of the cell groups is generated as shown in heatmap, either by a GFL or a non-parametric method. Estimated coefficients of this gene cluster using GFL inform the prior of hierarchical Bayesian model. Finally, *airpart* outputs estimates of allelic ratio for each gene and cell group, as well as *s*-value or adjusted *P*-value for AI and DAI test, respectively. Multiple visualizations of input data, gene clustering and fitted parameters are available as functions within *airpart* software

A summary of the *airpart* workflow is shown in Figure 1. *airpart* takes as input two count matrices and a categorical variable: (i) the allelic counts for the alternate (a1) and reference (a2) alleles across genes (rows) and cells/samples (columns) and (ii) the annotated cell types (or spatial location or time point for bulk RNA-seq) in the same order as cells/samples in count matrices. Annotation of cell type can either be provided as prior information or generated by clustering cells by total count [all single-cell RNA-sequencing (scRNA-seq) experiments analyzed here had prior annotation linking cells to their cell type]. The allelic counts could be generated using scBASE (Choi *et al.*, 2019), or the quantification pipeline outlined in Larsson *et al.* (2019) (e.g. for well-characterized diploid transcriptomes). Those inputs are used to construct a *SummarizedExperiment* (Lawrence *et al.*, 2013), and functions are provided to determine genes and cells passing quality control (QC). We define the observed allelic ratio as the count ratio of alternate allele reads to the total reads. *airpart* clusters genes with similar AI pattern across cells (see Supplementary Methods Section S1.1 for details). Clustering provides two benefits: it stabilizes DAI detection and estimation in the case that similar patterns occur across genes (e.g. genes under similar patterns of CTS *cis*-genetic regulation), and it speeds up computational time by fitting a partition model (described below) per cluster instead of per gene. In the following methods, we consider one gene cluster at a time. For each gene cluster, a GFL framework (Devriendt *et al.*, 2021) with Binomial likelihood is used to partition cell types, or a non-parametric method is used. Each of these relies on a graph Γ where vertices represent cell types and an edge indicates a pair of cell types that can be fused. *airpart* does not further partition cells within cell types, these are taken as fixed input of the method. DAI is declared if the partition has more than one group of cell types. Given the partition, a hierarchical Bayesian model is fit, which provides allelic ratio estimates and AI statistical inference. *airpart* also includes a number of visualization functions for exploratory data analysis, presentation of partitions and statistical inference on allelic ratios. A summary of the notation used in the following section is provided in Supplementary Table S1.

### 2.1 Distributional assumptions for allelic counts

In previous work, researchers often used a Binomial model (Castel *et al.*, 2020) or a Beta-Binomial (BB) model for the allelic counts (Castel *et al.*, 2015; Choi *et al.*, 2019; Edsgärd *et al.*, 2016; Heinen *et al.*, 2022; Santoni *et al.*, 2017; Skelly *et al.*, 2011; Zitovsky and Love, 2019), whereas BSCET uses a linear regression for the CTS AI test (Fan *et al.*, 2021). For the datasets examined in Section 3, either SMART-Seq2 single-cell datasets, or spatially or time-resolved bulk RNA-seq, we found that a Binomial assumption was sufficient for grouping cell types or conditions by AI, as many genes had minimal over-dispersion relative to a Binomial model. However, one real dataset examined in Section 3 exhibited over-dispersion of allelic counts relative to a Binomial, and so non-parametric methods were considered for the partition. When deriving per-gene allelic ratio estimates within a cluster, we modeled allelic counts using a BB generalized linear model (GLM). In summary, *airpart* offers Binomial or non-parametric models for partitioning cell types, and BB for deriving allelic ratio estimates.

### 2.2 GFL with binomial likelihood

*airpart* leverages a GFL framework (Devriendt *et al.*, 2021) implemented in an R package *smurf*, for partitioning cell types into groups of similar allelic ratio. For gene cluster *u*, suppose there are *G* genes, *I* cells and *K* cell types. For gene *g* and cell *i*, let *Y_gi_* indicates the observed allelic count for the alternative allele, *m_gi_* indicates total count, $r_{gi}=Y_{gi}/m_{gi}$ indicates the observed allelic ratio, *x_i_* indicates cell or sample state, which could be cell types, discrete spatial or time points and *c_i_* indicates any additional covariates that may associate with allelic ratios. We note that ***x*** and ***c*** are represented internally with dummy variables. We assume the following distribution for the alternative allele count with the logit link function

(1) $$Y_{gi}|m_{gi}∼\text{Bin}(m_{gi},p_{i}),$$

where *p_i_* indicates the true allelic ratio for cell *i*. We define $z_{i}=x_{i}{}^{T}\beta +c_{i}{}^{T}\gamma$, such that

(2) $$p_{i}=\frac{1}{1+e^{-z_{i}}}.$$

Without loss of generality, we will refer to the values of *x_i_* as cell types. The GFL in *smurf* is used to fit coefficients representing CTS allelic ratios, where fusing two coefficients means the two cell types are predicted to have a similar allelic ratio. Given the graph Γ as shown in Figure 1 in the Modeling panel, a complete graph (the default) can fuse all pairwise cell types differences, or alternatively, a flexible graph can be used with specific edges denoting the cell types that can be fused, e.g. an adjacency graph. The specific edges of Γ would be provided *a priori*, e.g. allowing fusing only among cell types on the same major branch of a developmental trajectory. Suppose h,k∈{1,…,K} are any two cell-type vertexes that are connected in the graph, then the regularized objective function for model is

$$\begin{array}{c} O(\beta ,\gamma ;x,m,r)=-\sum_{g,i}m_{gi}[r_{gi}z_{i}-\text{log}(1+e^{z_{i}})]+\lambda ||\Gamma (w)\beta || \end{array},$$

where the second part is the regularization term of the GFL (Höfling *et al.*, 2010). Here, *β_h_* and *β_k_* denote elements of full parameter vector β such that $(\beta_{0},\beta_{1},\ldots ,\beta_{K})=\beta$ and Γ(w) is the matrix with dimensions $N_{\Gamma }\times K$, where $N_{\Gamma }$ is the total number of edges in the graph Γ.

Estimation of coefficients relies on selection of optimal *λ* and specification of adaptive penalty weights for asymptotic consistency (Devriendt *et al.*, 2021). The standardized adaptive weights in *smurf* are defined as $w_{h,k}=\frac{K-1}{N_{\Gamma }}\sqrt{\frac{n_{h}+n_{k}}{N}}|{\hat{\beta }}_{h}-{\hat{\beta }}_{k}{|}^{-1}$ to adjust for possible level imbalances where *n_k_* represents number of cells in cell type *k*. *λ* is chosen according to the criterion of the lowest deviance (negative of log likelihood) within one standard error of the minimum deviance observed across a grid of *λ* values, based on 5-fold cross-validation, which encourages parsimony (more fusing of pairwise differences). When there are <8 cell types represented in ***x***, *λ* is chosen by finding the lowest deviance within half a standard error of the minimum, thus allowing more non-zero pairwise differences to persist. In addition to the Binomial likelihood (*airpart.bin*), a Gaussian likelihood (*airpart.gau*) is considered, which assumes $r_{gi}∼N(p_{i},\sigma )$. The different likelihood models for GFL were compared via simulation.

### 2.3 Pairwise Mann–Whitney–Wilcoxon test

An alternative method is considered and available within *airpart*, relying on a non-parametric test (*airpart.np*), both for increased speed and for cases when the distributional assumptions of the above model do not fit the data. We extend the Mann–Whitney–Wilcoxon (MWW) test to derive a partition based on the *P*-values from pairwise comparisons across cell types.

For all pairs of cell types with edges in Γ, pairwise MWW tests are performed for the allelic ratio distribution difference. A similarity score matrix S[K,K] is constructed, with elements equal to the MWW test *P*-values. Each element of this matrix is therefore related to the separability of the ranked allelic ratios for the two cell types. This matrix is then binarized into S′ as follows: $S\prime [h,k]=1_{S[h,k]<q}$ with 1 the indicator function. This binarization depends on a tuning parameter *q* and defines a network adjacency matrix. For pairs not represented by edges in Γ, S′[h,k] is set to 0. Finally, the adjacency matrix is used as a distance matrix for hierarchical clustering.

To choose the tuning parameter *q* and find the cell types partition, a model selection is performed based on the Bayesian information criterion (BIC). The BIC scores a candidate model using both its performance on the in-sample error and the complexity of the model. The best model is chosen by minimizing a loss function defined below, along a range of $q=10^{v}$, where default *v* sets are {−2,−1.8,…,−0.4}. The loss function is constructed based on the Gaussian special case of BIC that assumes independent errors from a normal distribution, and that the derivative of the log likelihood with respect to the true variance is zero (Hannan, 1982). We have

$$q=\underset{q}{\arg\min}\left[N\;\text{log}({\hat{\sigma }}_{e}^{2})+K_{q}\;\text{log}(N)\right],$$

where *N* is the total number of elements within this gene cluster (N=G×I), ${\hat{\sigma }}_{e}^{2}$ is an estimate of the error variance and *K_q_* is the number of groups derived from constructing the adjacency matrix according to each *q* threshold among the partition of *K* cell types. The estimate of the error variance in this case is defined as ${\hat{\sigma }}_{e}^{2}=\frac{1}{N}\Sigma_{g,i}{\left(r_{gi}-{\hat{r}}_{gi}\right)}^{2}$, which is a biased estimator for the true variance. In terms of partition group the loss function is

$$\begin{array}{c} q=\underset{q}{\arg\min}\left[N\;\text{log}\;\left(\frac{1}{N}\sum_{j=1}^{K_{q}}\sum_{i\in {\text{Grp}}_{j}}{\left(r_{i}-{\hat{r}}_{j}\right)}^{2}\right)+K_{q}\;\text{log}(N)\right] \end{array},$$

where ${\text{Grp}}_{j}$ is a set of cells in group *j*, ${\hat{r}}_{j}$ is the mean allelic ratio of all elements within group *j*.

### 2.4 Hierarchical Bayesian modeling

In the *airpart* steps to estimate the partition of cell types within a gene cluster, the true allelic ratio *p_i_* being modeled with the GFL is assumed not to vary across genes within the cluster. However, the allelic ratio may vary across genes within a cluster, though the clustering step brings together genes with similar patterns of allelic ratio. To derive per-gene allelic ratio estimates and credible intervals within a gene cluster, a Bayesian BB GLM is used. This GLM can be fit sharing prior information for all cells within a cell-type group defined by the partition (‘grouped’) or one cell type at a time, ignoring the partition (‘nogroup’). The case of ignoring the partition allows for estimation of allelic ratios even if the input ***x*** is continuous-valued. Let $\phi_{g}$ indicates a gene-specific dispersion parameter, then we assume the following model:

$$Y_{gi}|m_{gi}∼\text{BetaBin}(m_{gi},p\prime_{gi},\phi_{g}),$$

where $p\prime_{gi}={\left(1+\text{exp}(-x_{\mathrm{gi}}^{T}\beta \prime_{g}+c_{i}{}^{T}\hat{\gamma })\right)}^{-1}$, and $\hat{\gamma }$ is the maximum *a posteriori* estimate derived from *apeglm* (Zhu *et al.*, 2019; Zitovsky and Love, 2019) and used as offset in the model if covariates are provided. The dispersion $\phi_{g}$ controls the variance of *Y_gi_* by $\text{Var}(Y_{gi})=m_{gi}p\prime_{gi}(1-p\prime_{gi})\left[1+\frac{m_{gi}-1}{\phi_{g}+1}\right]$.

Shrinkage estimation is performed separately on the dispersion parameter and coefficients representing cell-type allelic ratios. We first describe shrinkage on the dispersion parameter. We assume the logarithm of the dispersion estimate ${\hat{\phi }}_{g}$ follows a Normal distribution,

$$\text{log}({\hat{\phi }}_{g})|\phi_{g}∼N(\text{log}(\phi_{g}),D),$$

where D, the sampling variance, is assumed equal across all genes in the cluster. We estimate this sampling variance with $\hat{D}=\frac{1}{G}\sum_{g}se_{g}^{2}$, where *se_g_* is the estimated standard error of the logarithm of the dispersion estimate $\text{log}({\hat{\phi }}_{g})$. Both $\text{log}({\hat{\phi }}_{g})$ and *se_g_* are estimated using the *apeglm* (Zhu *et al.*, 2019) software. Although the dispersion parameter is estimated per gene, the gene-wise models are linked by global hyper-parameters, which are estimated from the entire gene cluster at once. The specification of a cluster-specific prior is used a simple means of sharing information between genes. We assume that the dispersion parameter $\text{log}(\phi_{g})$ follows a Normal distribution

$$\text{log}(\phi_{g})∼N(\text{log}(\phi_{0}),A).$$

$\text{log}(\phi_{0})$ is estimated from the gene-wise MLEs, $\hat{\;\text{log}(\phi_{0})}=\frac{1}{G}\sum_{g}log({\hat{\phi }}_{g})$, and the variance is estimated with $\hat{A}=\max(s_{\;\text{log}\;\phi }^{2}-\hat{D},0)$, where $s_{\;\text{log}\;\phi }^{2}=\frac{1}{G-1}\sum_{g}(\text{log}({\hat{\phi }}_{g})-\hat{\;\text{log}(\phi_{0})}{)}^{2}$. In order to obtain the relative weighting of the gene-wise and global variance estimators, $B=\frac{D}{A+D}$ is defined as a parameter to shrink dispersion estimates toward a middle value [roughly following Efron and Morris (1975)]. Applying $\hat{B}=\frac{\hat{D}}{\hat{A}+\hat{D}}$, the final estimate for dispersion used in fitting coefficients is:

$$\text{log}({\hat{\phi }}_{g}^{\text{post}})=(1-\hat{B})\;\text{log}(\phi_{g})+\hat{B}\;\text{log}(\phi_{0}).$$

A Cauchy distribution is used as the prior for $\beta_{g}$, the coefficients representing cell-type allelic ratios. Shrinkage estimation is performed one group at a time, where a group is defined by the cell types within a partition from the first step or alternatively, ignoring groupings, meaning each cell type, temporal and spatial location is estimated by itself. Without loss of generality, we describe the grouped case. Let μ define the center of the prior distribution for $\beta_{g}$, which will be a vector of length *J* if there are *J* cell-type groups in this gene cluster. The GFL estimates $\hat{\beta }$ are used as $\hat{\mu }$ across the multiple genes within a cluster (or weighted means are used if non-parametric methods are used for defining the partition in the previous step). For the estimation of per-gene ratios, we assume the coefficients follow a Cauchy distribution,

$$\beta_{g}∼\text{Cauchy}(\mu ,S),$$

where ***S*** is scaling parameter estimated as part of the *apeglm* method (Zhu *et al.*, 2019), and $\hat{\mu }$ is plugged in as the center of the prior distribution. Maximum posterior estimates and credible intervals are estimated from a BB likelihood using *apeglm*.

### 2.5 Inference

To assess AI across each cell type and each gene within a cluster, *s*-values (Stephens, 2017) were calculated and provided, where thresholding on this value provides control of the aggregate false sign rate (the rate of incorrect signs of estimates within the reported set). For deriving inference of DAI calling, a likelihood ratio test was performed to compare a full model with a cell group indicator to a reduced model with an intercept only, whose test statistics approximates a $\chi^{2}$ random variable with J−1 degree of freedom if there are *J* cell-type groups in this gene cluster. We note that while the credible intervals, *s*-values and *P*-values calculated in this step reflect uncertainty in estimation of the allelic ratio based on number of cells and the range of the counts, as we fix the gene clustering and cell-type partition from previous steps, uncertainty from those upstream steps is not propagated to the inference provided by the hierarchical model.

### 2.6 Simulation setup

In order to assess *airpart’*s performance, partitioning of cell types by allelic ratio, and its accuracy of estimates of the allelic ratio itself, we performed three sets of simulation tests and compared to another statistical method for detecting heterogeneity of allelic ratio in scATAC-seq, *scDALI* (Heinen *et al.*, 2022). Various settings were summarized in Supplementary Table S2. The allelic counts were simulated from a BB distribution with constant dispersion parameter ϕ for all genes, so we ignore the index *g* here. The total counts were drawn from a Negative Binomial (NB) distribution. Half of the total counts had a mean count of two while half of the total counts had a higher mean count, ranging across different simulations among values of cnt∈{5,10,20}. In each case, the NB dispersion was set to *α* = 5 (dispersion *α* defined such that $\text{Var}(Y)=\mu +\alpha \mu^{2}$). As *airpart* combines allelic counts from multiple genes when finding the partition of cell types, having lower and higher total counts for each gene is equivalent to a dataset with a mix of low and high count genes within a gene cluster. The mean counts and BB dispersion values (ϕ=20) were chosen based on estimated parameters over real SMART-Seq2 scRNA-seq datasets (Larsson *et al.*, 2019), as shown in Supplementary Figure S1A. However, we observed that in earlier datasets, such as Deng *et al.* (2014), as shown in Supplementary Figure S1B, the allelic counts generated lower ϕ estimates (more variance). Thus, we also assessed simulations using ϕ=3 to evaluate method robustness when the data were substantially overdispersed relative to a Binomial model (the model used by the GFL in *airpart*). As *airpart* relies on cells being annotated upstream of its modeling steps, we additionally assessed modeling performance if the cells were incorrectly annotated [incorrect *t*(*i*) where *t* is the group] during clustering of sub-populations of cells by their total count. We constructed two scenarios to evaluate the robustness of partitioning and of allelic ratio estimation to cell annotation errors. In one scenario, we manually flipped cell-type labels to induce a fixed and uniform misclassification rate across all cell types. The misclassification rates ($\frac{1}{I}\sum_{i\leq I}\;1_{\left\{t(i)\neq x_{i}\right\}}$) were set to {0%,5%,10%} in this case. In the other, we clustered cells by log scaled total count with the specified cluster number of 10, allowing for random and heterogeneous misclassification rates across true cell type, as shown in Supplementary Figure S2. The misclassification rate was approximately equal to 5% using Gaussian Mixture Model (GMM) method implemented in *mclust* (Scrucca *et al.*, 2016) with the high mean total count cnt* *=* *7 for a subset of genes, and approximately equal to 10% using *k*-means implemented in *scran* (Lun *et al.*, 2016) and cnt* *=* *10 for a subset of genes. The two different clustering methods and varying the high total count parameter were used to help tune the misclassification rate to the desired level; additionally GMM clustering tended to give more uniform misclassification rate across clusters, while *k*-means clustering was more likely to give heterogeneous misclassification rates, e.g. entire cell types mis-labeled.

In the first set of simulations, the adjusted Rand index (ARI ∈[−1,1]) was used to assess *airpart’s* accuracy of the cell-type partitions with respect to the true partition by allelic ratio. The number of genes within a gene cluster was varied across g∈{5,10,20}. ARI of one means a perfect partition, and an ARI of zero is no better than random guessing. Each cell type was simulated to have 40 cells, and 10 cell types were simulated (400 cells in total) with true allelic ratio given by {0.95,0.9,0.85,0.85,0.7,0.7,0.65,0.6,0.5,0.5}, thus with a true partition of seven groups of unique allelic ratios. The whole simulation was repeated 200 times for each combination of simulation parameters: high total count (cnt), number of genes (*n*) and dispersion value (ϕ).

In the second set of simulations aiming for evaluating allelic ratio estimation accuracy, 400 genes were simulated such that all have the same pattern of DAI. The number of cells per cell type was varied from 40 to 100. Root Mean Square Error (RMSE) was used to evaluate performance. Here, we additionally assessed the effect of using the partition to aid in estimation accuracy, by comparing performance with and without this cell-type grouping step. The version of the estimation method without the grouping step was denoted as *airpart.nogroup*. We defined a simulation parameter d∈{0.05,0.1,0.2,0.3}, and at each *d*, allelic ratios according to {0.3,0.3,0.3+d,0.3+d,0.3+2d,0.3+2d,0.3+d,0.3+d} were simulated following a U-shaped pattern, where *d* controlled the extent of the rise and fall in allelic ratio. For this simulation, the cell-type indicator ***x*** was provided as a matrix with one-hot encoding to *airpart* and *scDALI*. We used *scDALI* with a radial basis function kernel to allow for cells with similar *x* value to have similar fitted allelic ratio, which in this case allows for estimation of CTS allelic ratios.

Lastly, in the third set of simulations for significance testing of DAI, genes were simulated without DAI (all six cell types with 0.5 ratio) and with DAI {0.5,0.5,0.6,0.6,0.7,0.7} with 40 and 100 cells per cell type. For *airpart*, a likelihood ratio test was performed to compare a full model with a cell-type indicator to a reduced model with an intercept only. *scDALI* was run with scDALI-Het to calculate score test statistics. We adjusted allelic heterogeneity *P*-values for both methods using the Benjamini–Hochberg (BH) (Benjamini and Hochberg, 1995) procedure with a cutoff of 0.05.

### 2.7 Allelic datasets

We applied *airpart* to two single-cell RNA-seq datasets: Larsson *et al.* (2019) and Deng *et al.* (2014); and two bulk RNA-seq datasets: Gutierrez-Arcelus *et al.* (2020) and Combs and Fraser (2018). From those datasets, Larsson *et al.* (2019) contains 224 mouse embryo stem cells (C57BL/6 × CAST/EiJ) and 188 mouse embryo fibroblasts (CAST/EiJ × C57BL/6J) grouped across states of cell cycle (G1, S, G2M), as identified by the authors. Deng *et al.* (2014) includes 286 pre-implantation mouse embryo cells composed of 10 cell types from an F1 cross of female CAST/EiJ and male C57BL/6J(B6) mice. Cells were sampled along a time course from the zygote and early two-cell stages through the late-blastocyst stage of development. Maternal allelic ratios were estimated for the two scRNA-seq datasets.

Gutierrez-Arcelus *et al.* (2020) stimulated memory CD4+ T-cells from 24 genotyped individuals of European ancestry with anti-CD3/CD28 beads and characterized the dynamics of AI events at 0, 2, 4, 8, 12, 24, 48 and 72 h after stimulation. Combs and Fraser (2018) performed RNA-seq of five hybrid *Drosophila melanogaster* × *Drosophila simulans* embryos sliced along their anterior–posterior axis to identify genes with spatially varying AI. Results applying *airpart* and *scDALI* to Combs and Fraser (2018) dataset are provided as Supplementary Results. The cell population annotations for all datasets were provided with the data. These annotations were used as known cell types/states/spatial position for analysis. The number of cells in Table 1 represents the size of each dataset after preprocessing (see Section 1.2 for details).

**Table 1.** Single-cell (sc) and bulk RNA-seq datasets used for evaluation

Source	Observations	States/contexts	Tissue type
Larsson *et al.* (sc)	367	4	Mouse F1 embryos
Deng *et al.* (sc)	228	10	Mouse F1 embryos
Gutierrez-Arcelus *et al.*	199^a^	8	Mem. CD4+ T-cells
Combs *et al.*	126^b^	19	Fly F1 embryos

a Time point replicates from 24 donors.

b Slices from five embryos.

To assess whether gene clusters with specific differential AI pattern detected by *airpart* were enriched for functional categories or were correlated with enhancer activity, we performed downstream functional analysis. Gene Ontology (GO) term (The Gene Ontology Consortium, 2020) enrichment was calculated using the *goseq* package (Young *et al.*, 2010) with the UCSC mm9 gene lengths database. Larsson *et al.* (2019) also provided H3K27ac ChIP-seq samples for one of the parental lines (B6) for mouse embryonic stem cells (mESCs) and fibroblasts. H3K27ac peaks were selected with fold enrichment >15. ChIP-seq was only available for the B6 strain, so we assessed whether the genes with AI toward one allele were more closely associated with enhancer activity in that cell type compared to the other cell type using a Fisher’s exact test.

## 3 Results

### 3.1 Simulation

**Fig. 2.**
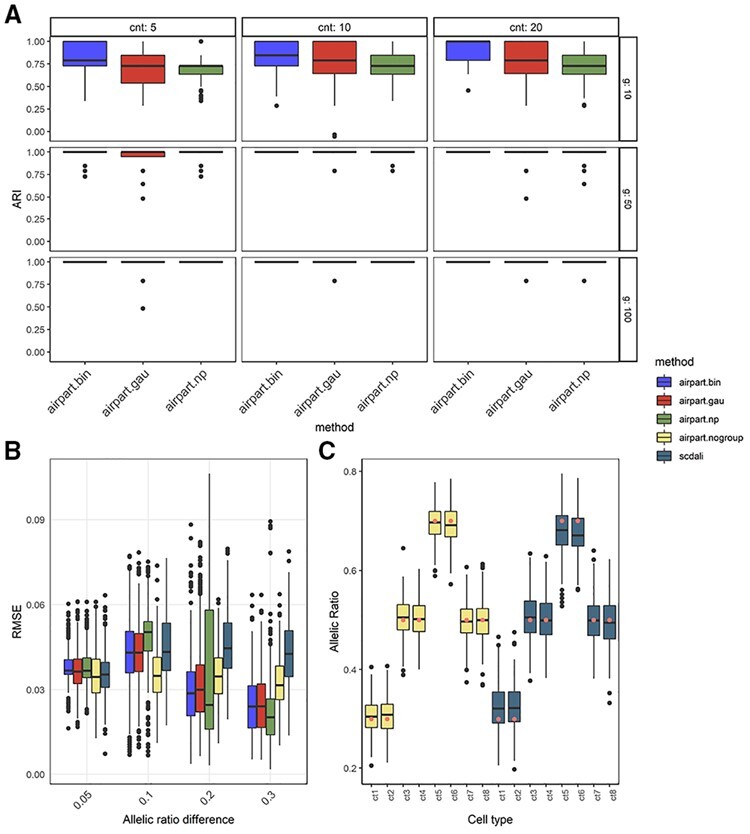
Performance comparison of *airpart* variants and *scDALI* on simulation datasets. (**A**) Boxplot of partition accuracy among three variants of *airpart*. *y*-axis is ARI among 200 iterations. cnt, the higher mean count; *n*, number of genes within a gene cluster. (**B**) Boxplot of RMSE per gene for estimation of the allelic ratio for *n *=* *40 cells among 400 iterations. Each gene has an underlying U-shape pattern described in the Section 2.6. (**C**) Boxplot demonstrating *airpart* without cell-type grouping step and *scDALI* performance on each cell type at DAI = 0.2. The highlighted dots inside the boxes represent the simulated allelic ratios

We evaluated *airpart* across a variety of simulation datasets, and in comparison to a newly developed method for detecting heterogeneous AI, *scDALI* (Heinen *et al.*, 2022). The simulated total counts distribution mimicked real scRNA-seq counts distribution (Supplementary Fig. S1C), and *airpart* clustered together genes for which the allelic ratio trend was similar (Supplementary Fig. S1D). On the simulated dataset with 10 cell types of different allelic ratio, as described in Section 2.6, the GFL with Binomial likelihood tended to have higher ARI than other variants when the number of genes within a gene cluster (*G*) was small (Fig. 2A). The higher ARI indicates that a method was more accurate at partitioning the cell types according to their true underlying allelic ratio. In addition, the GFL with Binomial likelihood had highest tolerance against uniform cell-type misclassfication errors (Supplementary Fig. S3). This aligned with previous work showing that modeling the allelic ratio using count distributions can increase power (Sun, 2012). The heterogeneous misclassification rate of ∼10% in some cases led to all cells of one cell type being misclassified, which further could result in the grouping step not achieving perfect recovery of the partitions (Supplementary Fig. S4). As all approaches could reach similar accuracy when *G* was larger than 25, we compared the computation time at that setting. The non-parametric method (*airpart.np*) was around 10 times faster than *airpart.bin* (Supplementary Fig. S5) and took 40s when *G *=* *500. We noticed that *airpart.np* could become overly sensitive to differences in allelic ratio when G was very large (≥350), such that when cell-type annotation was misclassified, this resulted in a decrease in ARI in particular relative to the GFL with Binomial likelihood which maintained perfect ARI (Supplementary Fig. S3). To consider robustness of *airpart* with respect to measurement error of an underlying expected allelic rate, we simulated counts with higher dispersion (ϕ=3). In these simulations, the ARI of all methods was generally lower especially for smaller number of genes, where the non-parametric approach outperformed the GFL alternatives (Supplementary Fig. S6).

We compared the allelic ratio estimation accuracy of *airpart* with or without grouping step to *scDALI* across different scales of DAI. *airpart.bin* had smaller RMSE than *scDALI* for allelic difference ≥0.2 while performing comparably for allelic difference of 0.1, and slightly worse for allelic difference of 0.05 (Fig. 2B). In this simulation, we assessed one gene at a time, so *airpart* did not benefit from aggregating signal across multiple genes. All three variants of *airpart* with partition step (Binomial likelihood, Gaussian likelihood and non-parametric approach) showed distinct decreasing RMSE with increasing allelic ratio difference, while *airpart.nogroup* (without cell-type partitioning) and *scDALI* had relatively constant RMSE. *airpart* variants with partition step benefited in this simulation from its approach toward discrete groupings of cell types, as the simulated data consisted of eight cell types falling in three groups by their true allelic ratio; as the allelic ratio increased, the correct partition was easier to identify, which led to the decrease in RMSE for those three method variants. In order to understand why *scDALI* tended to have slightly larger RMSE than *airpart.nogroup*, we examined the estimates themselves over the cell-type variable (***x***); *scDALI*’s estimates tended to shrink toward 0.5 at the extremes on this simulation (Fig. 2C). In the simulations with misclassified cells, *scDALI* had higher RMSE with increasing misclassification rate, while sharing information across cell types helped the *airpart* approaches to be less affected by the insertion of misclassified cells (Supplementary Fig. S7). From this simulation, we inferred that when the true model is one of discrete allelic ratios shared across a partition of the cell types, grouping cell types with similar allelic ratio increases observation size and may therefore aid in estimation.

We performed simulation with more cells per cell type (*n *=* *100 compared to *n *=* *40 in previous simulations) to confirm that estimates would have reduced error with more observations. Both *airpart* and *scDALI* had lower RMSE when *n *=* *100 (Supplementary Fig. S8A). *airpart.bin* additionally had better performance relative to *scDALI* for DAI =0.1, compared to the *n *=* *40 simulation. When considering credible interval coverage, *airpart.nogroup* had the highest empirical coverage (the average number of times the credible intervals contained the true value) almost always achieving 95%, although other *airpart* variants and *scDALI* performed as well when DAI > 0.1 (Supplementary Fig. S8B and C). Again note that *airpart* partitioned cell-type group per gene under this set up, so it did not benefit borrowing information from other genes with similar allelic pattern.

In the simulation assessing the rate of DAI calling when *n *=* *40, *airpart.nogroup* and *scDALI* both had the highest specificity of around 97% compared to other *airpart* variants (*airpart.bin* had 93.75%). But all methods had similar sensitivity of around 98.00% (Supplementary Table S3). For *n *=* *100, *airpart.bin* had the highest specificity of 98.25%, likely due to its increasing accuracy in determining the partition of cell types (Supplementary Table S4). *scDALI* and other variants’ specificity did not change substantially as *n* increased to 100, but all methods had 100% sensitivity at this sample size. *aipart.gau* and *airpart.np* had lower specificity in this global test of DAI (89.5% and 88.0% at *n *=* *40, and 85.25% and 88.25% at *n *=* *100), which was expected as these variants often detected too many groups in the partition analysis for less overdispersed data, with lower ARI relative to *airpart.bin* (Fig. 2A). Overall, *airpart.bin*, *airpart.nogroup* and *scDALI* recovered most DAI while not falsely calling too many genes as DAI. We expect *airpart* would have improved performance when adding a gene clustering step, such that it can borrow information about cell-type partitioning and allelic ratio estimation across genes.

In summary, we recognize that *airpart* and *scDALI* have subtly different inference goals, with *airpart* predominantly focused on characterizing the allelic ratio patterns that result from discrete groups of cell types sharing a common regulatory context (e.g. expression of transcription factors and accessibility of CRE). On the other hand, *scDALI* is more suitable for detecting various types of heterogeneous AI including continuous gradients of AI in cells across measured or inferred dimensions.

### 3.2 Mouse ES cells and fibroblasts

For assessing *airpart* on real allelic datasets, we first examined two single-cell RNA-seq datasets consisting of mouse embryo cells (Deng *et al.*, 2014; Larsson *et al.*, 2019). Both datasets were mouse F1 non-reciprocal crosses in which we observed clusters with AI toward the maternal allele, likely from imprinting in mature cells or genome activation for early cell stages. A complete graph was applied to both datasets, allowing any developmental time point coefficients to be fused with another. In both cases, *airpart* partitioned the cell stages as expected according to developmental time, e.g. consecutive and related time periods being fused together, such as early, mid and late-blastocyst.

**Fig. 3.**
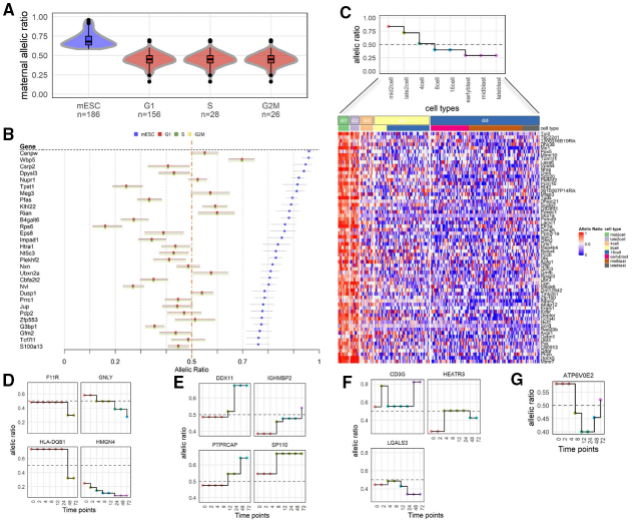
Evaluation of airpart on two scRNA-seq experiments. (**A**) Violin plot of estimated allelic ratio on Larsson’s dataset with *n* indicating the number of cells. Color represents different partition groups. (**B**) Forest plot for Larsson’s dataset, showing top 40 genes with smallest *s*-value. Dotted line denotes allelic ratio = 0.5 (**C**) Step plot and heatmap of results for Deng’s dataset. This gene cluster partitioned cell types into five groups denoted by highlighted dots in the step plot. (**D–G**) Selected genes displaying *airpart* fitted model on Gutierrez-Arcelus’s data: (D) decreasing trend, (E) increasing trend, (F) up-down pattern and (G) down-up pattern

*airpart* was first applied to the Larsson *et al.* (2019) dataset consisting of four cell states including three cell cycle states of primary mouse fibroblast (G1, S and G2M) and mESCs. After QC filtering, 2481 genes remained and five gene clusters were detected. Four of the five clusters, comprising 412 genes in total, showed evidence of DAI by their *airpart* partitions (Supplementary Fig. S9A and Supplementary Table S5). One cluster of 128 genes partitioned the cell states such that all cell cycles of fibroblast were grouped together and apart from the mESC (Fig. 3A and Supplementary Fig. S9C). In this cluster, the fibroblast group had mean estimated allelic ratio around 0.45 and the mESC had AI with a ratio of around 0.70 toward the maternal allele. We estimated the AI in both mESC and fibroblast and calculated 95% credible intervals (Fig. 3B). With an *s*-value threshold of 0.005 (Section 2.4), all 128 genes demonstrated AI in mESCs and 85 genes out of 128 demonstrated AI in fibroblasts, which was roughly consistent with credible intervals not overlapping an allelic ratio of 0.5. To check whether this cluster had any functional association with stem cell maintenance, gene set enrichment analysis was performed. The most significant GO term was ‘response to leukemia inhibitory factor (LIF)’ [GO: 1990823, odds ratio = 4.90, adj. *P* =0.136 (BH)], where *Lif* is a cytokine involved in embryonic stem cell self-renewal (Hirai *et al.*, 2011).

To assess whether the 128 gene cluster with DAI had an association with enhancer activity, an enrichment analysis was performed using H3K27ac peaks exclusively measured in the B6 strain in both cell types. While the enhancer activity signal was therefore not an allelic signal, we hypothesized that genes which favor the B6 (maternal) allele in mESC may tend to overlap with H3K27ac peaks in B6 in mESC. For the 128 gene cluster with DAI toward maternal allele in mESC, we found that genes in the cluster were more often associated with enhancer activity in mESC compared to fibroblast (significance assessed by Fisher’s exact test *P *=* *0.0002).

*airpart* was additionally applied to the Deng *et al.* (2014) dataset consisting of 228 pre-implantation mouse embryo cells passing our QC steps, from an F1 cross of CAST/EiJ × C57BL/6J mice. A total of 10 cell types were annotated from the zygote and early two-cell stages through the late-blastocyst stage of development. Allelic ratio was defined as maternal [CAST/(B6 + CAST)] and 4679 genes passed our gene QC steps. All genes revealed DAI patterns as expected due to genome activation from zygote onward to the blastocyst stages. In order to focus on DAI after zygote or early two-cell stages, we performed clustering of genes and partitioning of cell stages with those cell stages removed. A total of 13 out of 16 gene clusters, consisting of 3019 genes in total, showed DAI pattern after removing these cell stages (Supplementary Table S6). One gene cluster showed a decreasing allelic ratio pattern along developmental time. We demonstrated benefits of *airpart* modeling, estimating each gene’s allelic ratio leveraging the gene cluster’s GFL coefficients as a prior mean (Fig. 3C). The corresponding violin plot of allelic ratios is shown in Supplementary Figure S9C. The estimated allelic ratio around 0.3 in early/mid/late blast stage was an exception (for most gene clusters, blastocyst cells showed near to balanced AE). This cluster of 65 genes had significant enrichment for GO terms, such as ‘cell development’ [GO: 0006139, odds ratio = 2.02, adj. *P* =0.0017 (BH)] and ‘cell differentiation’ [GO: 0030154, odds ratio = 1.29, adj. *P* =0.0233 (BH)].

### 3.3 Dynamic AI during T-cell activation

We applied *airpart* to an RNA-seq dataset of stimulated memory CD4+ T-cells of eight discrete time points (Gutierrez-Arcelus *et al.*, 2020). To do so, we created a graph Γ with edges only between consecutive time points, restricting the fusion of coefficients in the GFL. Among the 43 most temporal-varying genes as described in Supplementary Section 1.2, most of them were enriched within autoimmune loci, as reported by the original study authors. Examples include *F11R*, a ligand for integrin alpha-L/beta-2 involved in memory T-cell and *HLA-DQB1*, a member of the HLA complex. We ignored the allelic complexity of the HLA genes in this analysis and grouped the alleles together into two, based upon the SNP with the largest total count. We chose to reduce to diploid allelic counts in each individual based on a single SNP since HLA typing and across-donor inference of more than two alleles was out of the scope of this study. This approach was used for method demonstration only. *airpart* partitioning of the time series by allelic ratio revealed four types of patterns (decreasing, increasing, up-peak and down-peak) as shown in Figure 3(D–G), respectively (step-plots for all 40 genes provided in Supplementary Fig. S12). As in the original study, we also observed the dominant allele could switch over the time course, or bi-allelically expressed genes could switch to dominant by one or the other allele. While the original paper used logistic regression with polynomial terms for time within each individual, we recovered similar DAI trends for many autoimmune genes, such as *GNLY* and *DDX11*. Overall, *airpart* successfully captured the DAI patterns seen across T-cell activation.

In summary, when applied to scRNA-seq and bulk RNA-seq datasets, *airpart* was able to identify relevant partitions of cell types or samples, with gene clusters significantly enriched for biologically meaningful gene sets and CTS enhancer activity. Results applying *airpart* and *scDALI* to a dataset of spatial transcriptomic fly cross embryos [Combs and Fraser (2018)] are provided in Supplementary Section 2.1, where airpart with basis matrix was used to estimate smooth fitted ratios for genes with spatially varying AI induced by continuous gradients of regulatory proteins.

## 4 Discussion

An understanding of how individual genes may be regulated across context or condition helps to elucidate molecular mechanisms underlying complex phenotypes or diseases. Context-specific AE enables isolation of *cis*-acting genetic regulation of transcription, and the study of AE is a good complement to differential gene expression studies, where a multitude of factors may influence differences in total expression across condition. Single-cell RNA-seq of F1 crosses enables measurement of context-specific AE, where the cell type or cell stage can be taken as the context that influences *cis*-genetic regulation. Spatially resolved or time course allelic datasets offer another such example. Context-specific or conditional allele-specific expression datasets can detect AI with fewer samples than context-specific quantitative trait locus studies (Findley *et al.*, 2021), although measurement of allele-specific expression in a sample requires presence of heterozygous variation in the transcribed region, which may not occur for all transcripts or for all genes depending on the population under study. With the advent of large-scale systematic assays for interrogating variants and regulatory elements, such as CRISPR-Cas9 and massively parallel reporter assays, there are now increasing opportunities to re-use context-specific allelic datasets, which can help point to the key cell types or cell states for validation.

To assist with analysis of such datasets, we developed *airpart*, a statistical framework for identifying genes and cell types or cell stages with similar DAI signal. *airpart* provides discrete grouping of cell types, providing interpretability to the fitted models. The groups provided by the partition step can help to generate hypotheses of CTS *cis*-regulatory mechanisms. For example, cell types within the same group may share a common mechanism of *cis*-regulation, such as a common set of expressed transcription factors and active regulatory elements harboring genetic variation. Taken together, our simulation results suggest that *airpart.bin* (using a Binomial likelihood) had good performance across a variety of number of cells and genes, including when cells are misclassified, and can be used for accurate cell-type grouping when the counts are not highly overdispersed, as was observed in more recent SMART-Seq datasets. When the aim is allelic ratio estimation or overall DAI hypothesis testing, *airpart.np* or *airpart.nogroup* may be preferred for faster computation time and comparable accuracy to *airpart.bin*. As scRNA-seq data often have low counts for some genes of interest, and as the experiments used for AE in single cell (SMART-Seq2 or SMART-Seq3) often have a relatively small number of cells per donor, we simulated *I *=* *400 cells (40 cells per cell type × 10 cell types) to be comparable, and employed gene clustering and a partitioning of cell types in order to increase power. Aside from using clustering to detect meaningful subsets of genes by AI, it is also possible to provide pathways or other gene sets known *a priori* for *airpart* to partition. In this way, *airpart* stabilizes gene-level estimation by borrowing information about the similarity of cell types from other genes that have similar AE patterns.

*airpart* can be applied to a variety of problems, as it leverages a GFL framework (Devriendt *et al.*, 2021) where a graph specifying the connectivity of the cell types is provided, helpful for scenarios, such as time course experiments or for prohibiting fusing across different cell lineages. Another point of flexibility is *airpart’*s use of a design matrix within the GLM [Equation (2)] wherein additional covariates can be provided that may also have effects on the allelic ratio. This was used here in the analysis of the time course RNA-seq dataset to adjust for individual effects, and may be helpful for multi-individual single-cell sequencing studies. Furthermore, *airpart.nogroup* can accept a design matrix representing natural cubic splines. *airpart* therefore offers fast estimation of smooth functions of the allelic ratio over a continuous variable, making use of a hierarchical model to stabilize the over-dispersion parameter (Supplementary Section 2.1). Though *airpart* predominantly focuses on characterizing the allelic ratio patterns that result from discrete groups of cell types under shared regulatory contexts, *airpart* can in this way be used to model continuous gradients of *cis*-regulatory effects on cells or samples.

## Data availability

*airpart* is implemented as an R/Bioconductor package available at: https://bioconductor.org/packages/airpart. All of the R code and data used in this article for evaluating methods on simulated and real RNA-seq datasets are available at the following repository: https://github.com/Wancen/airpartpaper.

## Supplementary Material

btac212_Supplementary_Data
